# Can tempo-based strength periodization training improve performance in coastal rowers? A 14-week longitudinal study

**DOI:** 10.7717/peerj.21376

**Published:** 2026-06-18

**Authors:** Mushuai Hao, Haonan Qi, Hong Qin, Liang Zhao, Haitao Han, Wei Han

**Affiliations:** 1 School of Competitive Sports, Shandong Sport University, Rizhao, Shandong Province, China; 2 College of Physical Education, Hebei Normal University, Shijiazhuang, Hebei, China; 3 School of Physical Education and Health, Hunan University of Technology and Business, Changsha, Hunan, China; 4 Shandong Provincial Sports Bureau, Shandong Sports Science Research Center, Jinan City, Shandong Province, China

**Keywords:** Strength periodization, Movement tempo, Coastal rowing, Athletic performance

## Abstract

**Objective:**

This study aimed to investigate the effects of a 14-week tempo-based strength periodization training program on muscle strength, power, and sport-specific performance in coastal rowers.

**Design:**

A single-group pre-post study design was implemented.

**Method:**

Twelve well-trained coastal rowers (age 20 ± 2.34 years; height 182.42 ± 4.83 cm; weight 79.25 ± 10.17 kg; training experience 6.33 ± 2.81 years) underwent a 14-week periodized training program, which consisted of four phases: hypertrophy, transition, maximal strength, and tapering. Three testing time points were set: baseline (T1), post-hypertrophy (T2), and post-intervention (T3). Assessments included maximal strength (1-repetition maximum squat, bench press, bench pull, deadlift), power (countermovement jump height and peak power), and sport-specific performance tests (50 m sprint, 500 m ergometer, and composite test: 50 m sprint + 750 m ergometer + 50 m sprint).

**Results:**

Significant improvements were observed from T1 to T3 in maximal strength (squat: *P* < 0.001, Effect Size (ES) = 1.01; bench press: *P* < 0.001, ES = 0.86; bench pull: *P* < 0.001, ES = 1.06; deadlift: *P* < 0.001, ES = 0.93), countermovement jump (CMJ) height (*P* < 0.001, ES = 0.37), 50-m sprint (*P* < 0.001, ES = –0.42), 500-m ergometer performance (*P* < 0.001, ES = –0.49), and the composite test (*P* < 0.001, ES = –1.1). No significant change was found in CMJ peak power (*P* > 0.05, ES = 0.16).

**Conclusions:**

A 14-week tempo-based strength periodization program was associated with significant improvements in muscle strength, countermovement jump (CMJ) height, and sport-specific performance in coastal rowers, suggesting potential utility in integrating structured tempo training into periodized strength plans for power-dependent water sports athletes.

## Introduction

The International Olympic Committee’s official inclusion of coastal rowing beach sprint in the Los Angeles 2028 Olympic programme in April 2025 marked its establishment alongside traditional rowing as a significant component of the Olympic schedule. Coastal rowing beach sprint exhibits distinct observed competitive characteristics relative to traditional flat rowing: events are brief (approximately 3 min for single sculls), high-intensity, and combine beach sprinting, open-water rowing, and technical buoy turns, which impose unique and substantial physical demands on athletes. Moreover, the unpredictable wind and wave conditions in open water present substantial environmental challenges that necessitate adequate physical strength and postural stability. Consequently, identifying effective methods to enhance the strength and performance of coastal rowers has become a primary concern for practitioners in this field.

In high-performance training, periodization provides the essential framework for organizing training loads and facilitating peak performance (Hartmann et al., 2015). While various models exist and debates continue regarding their respective merits (*e.g.*, traditional *versus* block periodization) (Stone et al., 2021; Bazyler et al., 2015; Mujika et al., 2018), the underlying principle involves the coordinated development of different athletic qualities to enable athletes to peak at major competitions. A typical strength training periodization model often comprises phases such as anatomical adaptation, hypertrophy, maximum strength, conversion, and maintenance (Bompa & Carko, 2019). Within this framework, over the years, extensive research has been accumulated in the field of physical training for traditional rowing (Nugent et al., 2020; Ledergerber et al., 2023; Behm, Jacobs & Schumann, 2025; Thiele et al., 2020). However, as an emerging Olympic discipline, coastal rowing still lacks long-term exploratory research in this domain.

Given that coastal rowing integrates distinct phases such as beach sprints and open-water rowing, it imposes unique demands on the muscle’s eccentric-isometric-concentric sequential force production, which differ significantly from those of traditional rowing. Currently, the design of strength training programs tailored to this specific force production requirement, along with corresponding empirical investigations, remains a critical gap in the scientific literature. Irrespective of the periodization model, the efficacy of the training methods employed is paramount. In recent years, controlling movement tempo has gained increasing attention as a key training variable (Adrián, José & Markel, 2021; Shibata et al., 2021; Stasinaki et al., 2019). Movement tempo refers to the speed of each repetition during resistance training, often prescribed using a numerical sequence (*e.g.*, 4/0/1/0 denoting a 4-second eccentric phase, no isometric pause, and a 1-second concentric phase), aimed at optimizing neuromuscular adaptations and force-time characteristics. Within such notation, ‘X’ often signifies an explosive tempo, while ‘V’ indicates a voluntary velocity (Michal, Tufano & Adam, 2020). Most existing empirical studies on movement tempo represent general tempo manipulation that focuses exclusively on controlling the velocity of only a single contraction phase (eccentric or concentric) (Pearson et al., 2022; Pereira et al., 2016; Segers et al., 2022; Walker et al., 2020), rather than a structured, sequential tempo across the full stretch-shortening cycle. For example, prior work has compared the effects of different eccentric tempos on hypertrophy (Pearson et al., 2022; Pereira et al., 2016) or analysing the role of eccentric duration on power output (Segers et al., 2022). Such single-phase tempo interventions, as widely adopted in Shibata et al. (2021), Stasinaki et al. (2019), Michal, Tufano & Adam (2020), Pearson et al. (2022), Pereira et al. (2016) and Segers et al. (2022), are limited to isolated modulation of one contraction mode, rather than a coordinated, phase-integrated tempo sequence. Phased training approaches that systematically integrate eccentric-isometric-concentric elements remain scarce.

Owing to variations in interventions (often manipulating a single tempo component), subject populations (rarely involving trained athletes) (Nóbrega et al., 2018; Brien, Browne & Earls, 2020), and outcome measures across existing studies, it is difficult to establish an evidence-based movement tempo prescription for athletic populations. Moreover, an isolated focus on a single contraction phase fails to replicate the integrated “eccentric-isometric-concentric” sequence characteristic of sports movements, appearing oversimplified for specific performance contexts. The Triphasic Training system, proposed by Dietz & Peterson (2012), offers a highly structured framework for applying movement tempo. Rooted in the residual training effects theory associated with block periodization, it divides training into accumulation, transmutation, and realisation blocks. The accumulation block is further subdivided into distinct eccentric, isometric, and concentric phases, each prescribed with specific loads and movement tempos (*e.g.*, slow eccentric, isometric pause, accelerated concentric).

The core premise is that the nervous system adapts specifically to different contraction modes, and thus training each phase systematically enhances overall movement force production (Dietz & Peterson, 2012). Although this theory requires further scientific validation, its structured emphasis on movement tempo provides a valuable rationale for designing training interventions.

Given the specific strength demands of coastal rowing and the potential theoretical benefits of movement tempo training, this study adopted the phased tempo control principles of the accumulation block from the Triphasic Training model, adapting it to the sport’s demands to design and implement a 14-week strength training mesocycle.

Critical knowledge gaps persist in the field: while movement tempo manipulation has been explored in resistance training (Adrián, José & Markel, 2021; Shibata et al., 2021; Stasinaki et al., 2019), existing studies rarely integrate Triphasic-inspired “eccentric-isometric-concentric” tempo sequencing, nor have they applied such structured training to elite coastal rowers or verified its transfer to the sport’s specific performance outcomes. Addressing these gaps, this study aimed to explore the adaptive responses of muscle strength, CMJ height, and sport-specific performance in elite coastal rowers to periodized strength plan emphasizing movement tempo control, and to generate applied empirical data for the strength training of this emerging olympic sport. We hypothesised that participation in this training intervention would be associated with significant improvements in athletes’ maximal strength, power, and specific race performance.

## Methods

### Experimental approach to the problem

This study utilized a one-way repeated measures analysis of variance (ANOVA) design to examine training adaptations in coastal rowers over a 14-week mesocycle training intervention. All training and testing sessions were conducted at the facilities of a provincial-level sports team. The assessments included measures of maximal strength, 50-m sprint performance, countermovement jump (CMJ) height and peak power, 500-m ergometer rowing performance, and a composite test (50-m sprint + 500-m ergometer + 50-m sprint). Testing was performed at three time points: one week before the training program initiation (T1: pre-training), after the hypertrophy phase (T2: week 7), and after the maximal strength phase (which incorporated tempo-based training) (T3: week 15). The testing sequence was identical across all time points, with 24–48 h of rest between each testing day to minimize fatigue.

Specifically, the testing protocol was structured as follows:

Day 1: Maximal strength tests, including one-repetition maximum (1RM) assessments for the squat, bench press, deadlift, and bench pull.

Day 2: CMJ tests to evaluate jump height and peak power output.

Day 3: 50-m sprint test and 500-m ergometer rowing test.

Day 4: Composite test consisting of a 50-m run + 750-m ergometer row + 50-m run.

Throughout all testing sessions, the head coach, assistant coaches, and strength and conditioning coach were present to ensure standardization and adherence to protocols. Data collection and statistical analyses were managed by the strength and conditioning coach to maintain consistency and accuracy.

A single-group pre-post design was adopted due to the practical constraints of elite sports team training; isolating athletes into a control group would have disrupted the unified winter training plan and compromised ecological validity.

### Subjects

A total of 12 well-trained athletes (age: 20 ± 2.34 years; height: 182.42 ± 4.83 cm; body mass: 79.25 ± 10.17 kg; training experience: 6.33 ± 2.81 years) participated in this study. Among them, three were national team members, two were national-level masters of sports, and the remainder were first-class athletes. All athletes met the following inclusion criteria:

(1) Engaged in systematic training for more than 10 sessions per week (excluding morning training).

(2) Free from orthopedic or neurological disorders.

(3) No use of any drugs or banned substances.

Prior to testing, all athletes were informed of the experimental procedures and provided written informed consent. The study protocol adhered to the principles of the Helsinki Declaration (1975, revised in 2000) and was approved by the Ethics Committee of Shandong Sport University (Approval No. 2024068).

### Procedures

All training sessions were conducted in a professional fitness facility. Athletes followed a structured training program designed by the coaching team (specific program details are presented in Table 1).

**Table 1:** Overall design of strength program.

Training phase	Week	Structural exercises sets × Reps (%1RM)	Accessory exercises (Kettlebells, Dumbbells, Machines, *etc.*) Sets × Reps	Olympic lifts sets × Reps (%1RM)
Hypertrophy Phase	0	Baseline Testing (T1)		
	1	4 ×12 (65%–70%1RM)	3 ×12	3 ×10 (60%1RM)
	2	4 ×12 (65%–70%1RM)	3 ×12	3 ×10 (60%1RM)
	3	5 ×12 (65%–70%1RM)	4 ×12	4 ×10 (60%1RM)
	4	5 ×12 (65%–70%1RM)	4 ×12^*^	4 ×10 (60%1RM)
	5	6 ×12 (65%–70%1RM)	5 ×12^*^	4 ×10 (60%1RM)
	6	6 ×12 (65%–70%1RM)	5 ×12^*^	3 ×6 (70%1RM)
Transition Week	7	Mid-term Testing (T2)		
Maximal Strength Phase (Triphasic)	8	Mon, Tue: 5 ×5 (75%1RM) Thu, Fri: 5 ×3 (90%1RM)	4 ×8	4 ×6 (70%1RM)
	9	Mon, Tue: 5 ×5 (75%1RM) Thu, Fri: 5 ×3 (90%1RM)	4 ×8	4 ×5 (75%1RM)
	10	Mon, Tue: 5 ×5 (75%1RM) Thu, Fri: 5 ×3 (90%1RM)	4 ×8	4 ×5 (75%1RM)
	11	Mon, Tue: 5 ×5 (75%1RM) Thu, Fri: 5 ×3 (90%1RM)	4 ×8	4 ×4 (75%–80%1RM)
	12	Mon, Tue: 5 ×5 (75%1RM) Thu, Fri: 5 ×3 (90%1RM)	4 ×8	4 ×4 (75%–80%1RM)
	13	Mon, Tue: 5 ×5 (80–85%1RM) Thu, Fri: 5 ×1-3 (90–100%1RM)	4 ×8	4 ×3 (80%–85%1RM)
	14	Mon, Tue: 5 ×5 (80–85%1RM) Thu, Fri: 5 ×1-3 (90–100%1RM)	4 ×8	4 ×3 (80%–85%1RM)
Testing Week	15	Post-testing (T3)		

**Notes.**

* Special training methods were employed (*e.g.*, drop sets, super sets, giant sets) to reduce monotony and increase training volume.

### Training intervention

All athletes underwent an identical periodized training program, which was divided into four distinct phases:

1. Hypertrophy phase (Weeks 0–6)

Focused on muscle growth through high-volume resistance training.

2. Transition week (Week 7)

Served as a bridge to the maximal strength phase, involving load adjustments and recovery.

3. Maximal strength phase (Weeks 8–14)

Weeks 8–9: Eccentric contraction phase, emphasizing slow eccentric movements.

Weeks 10–11: Isometric contraction phase, incorporating pauses and holds.

Weeks 12–14: Concentric contraction phase, focusing on explosive concentric actions.

4. Tapering Week (Week 15)

Dedicated to testing and recovery, with reduced volume to optimize performance assessment.

Strength training was scheduled four times weekly (on Mondays, Tuesdays, Thursdays, and Fridays), with the overall structure detailed in Table 1 and phase-specific movement tempo requirements specified in Table 2. The strength training schedule featured two blocks of consecutive training days, separated by a 48-hour active recovery period on Wednesdays, with no strength training sessions scheduled on Saturdays and Sundays to facilitate neuromuscular recovery from resistance exercise.

**Table 2:** Tempo prescription.

Training Phase	Week	Movement Tempo
Hypertrophy Phase	0	Baseline Testing (T1)
	1	No specific movement tempo requirements
	2	
	3	
	4	
	5	
	6	
Transition Week	7	Mid-term Testing (T2)
Maximal Strength Phase (Triphasic)	8	Eccentric Tempo Week Mon, Tue:3/0/X Thu, Fri:X/X/X
	9	
	10	Isometric Tempo Week Mon, Tue:X/2/X Thu, Fri:X/X/X
	11	
	12	Explosive Tempo Week Mon, Tue:X/XX Thu, Fri:X/X/X
	13	
	14	
	15	Post-testing (T3)

**Notes.**

Notes: 3/0/X notation: The first digit = eccentric tempo, second = isometric tempo, third = concentric tempo. For 3/0/X: 3s eccentric, no isometric pause, maximal concentric tempo.

X = explosive tempo.

During the winter training phase, due to environmental constraints, on-water training was conducted in still water instead of open water, with a focus on technical buoy turning drills (no distance metrics were recorded for these skill-oriented exercises).

Concurrent ergometer training intensity was standardized using heart rate zones (target: 65%–80% of maximum heart rate) and total training duration, rather than distance-based metrics. All athletes adhered to the same weekly training structure (frequency, duration, and intensity ranges) to minimize confounding variables, ensuring consistency of the training intervention.

Load adjustment criteria were differentiated based on exercise type and training objectives. For all tempo-controlled core exercises (*e.g.*, back squat, bench press, lying pull), a “standardized baseline with dynamic adjustment” protocol was implemented: a minimum load threshold (≥ 80% 1RM) was predefined by the coaching staff, and athletes made individual micro-adjustments (±5% to 10%) based on their daily physical and mental status. For training content involving explosive contraction tempos and key auxiliary exercises (*e.g.*, front squat), load progression strictly followed the National Strength and Conditioning Association (NSCA)-recommended “2-for-2 rule”: if an athlete completed two additional repetitions with proper technique in the final set of two consecutive training sessions, the load was increased by 5–10 kg in the subsequent session. For remaining auxiliary exercises, load intensity was self-regulated by the athletes, with training termination defined as the attainment of technical failure (*i.e.,* the inability to complete an additional valid repetition due to movement form degradation).

### Maximal strength testing protocol

Maximal strength testing was conducted for the squat, deadlift, bench press, and bench pull exercises, following the standardized procedures recommended by the National Strength and Conditioning Association (NSCA). Prior to testing, the strength and conditioning coach explained the entire protocol to all athletes and displayed it on a training board for reference. The testing protocol involved the following steps:

1. Warm-up: After a general warm-up, athletes performed a set with a load that could be comfortably lifted for 10 repetitions.

2. Rest: 1 min of rest was provided.

3. Initial load estimation: A warm-up load was estimated for 3–5 repetition maximum (RM) using the following increments:

Upper-body exercises (bench press, bench pull): 5–9 kg or 5%–10% of estimated 1RM.

Lower-body exercises (squat, deadlift): 14–18 kg or 10%–20% of estimated 1RM.

4. Rest: 2 min of rest was allowed.

5. Submaximal load estimation: A conservative load for 2–3 RM was estimated using the same increments as above.

6. Rest: 2–4 min of rest was provided.

7. Progressive loading: The load was increased using the same increments (upper-body:

5–9 kg or 5%–10%; lower-body: 14–18 kg or 10%–20%).

8. 1RM attempt: Athletes attempted a 1RM lift. If successful, they rested for 2–4 min and returned to Step 7 for further loading. If failed, they rested for 2–4 min, and the load was reduced by:

Upper-body exercises: 2–5 kg or 2.5%–5%.

Lower-body exercises: 7–9 kg or 5%–10%.

The process was repeated from Step 8 until the athlete could complete one repetition with proper technique. The goal was to determine 1RM within 3–5 attempts.

Due to the athletes’ extensive strength training experience (mean training years: 6.33 ± 2.81), they self-selected loads for initial estimations. During formal 1RM testing, the strength and conditioning coach supervised each attempt to ensure technique safety and provided recommendations for subsequent attempts based on performance.

### Power testing: CMJ protocol

Countermovement jump (CMJ) height and peak power were assessed using a linear position transducer (GymAware; Kinetic Performance Technologies, Canberra, Australia). The testing procedure was as follows:

Setup: The GymAware system and its tablet application were activated. The “squat jump” mode was selected, and the athlete’s body mass was entered into the app. The transducer cable was attached to one end of a PVC pipe, which the athlete placed across the upper trapezius with hands relaxed to secure it.

Calibration: The app was used to zero the position of the PVC pipe while the athlete stood motionless.

Testing: Athletes were instructed to perform a maximal vertical jump without excessive forward or backward movement. The strength and conditioning coach monitored each jump to ensure compliance.

Trials and rest: Three trials were performed, with the average value used for analysis.

Each jump was separated by 1 min of rest to allow full recovery.

### Sport-specific performance testing protocol

This test is designed to evaluate the comprehensive performance of coastal rowing athletes, consisting of three sections as follows:

Part A: 50-m Sprint test

Athletes performed three maximal 50-m straight sprints, with a 5-minute recovery interval between each sprint.

Specific procedures

1. A timer, positioned at the finish line, operated a Seiko stopwatch.

2. A starter, standing at the starting line, held a flag.

3. The starter raised the flag to signal preparation.

4. The starter called out loudly and clearly “On your marks–Go” while lowering the raised arm; the stopwatch at the 50-meter finish line was activated simultaneously when the arm was lowered.

5. The time was recorded when the athlete’s chest crossed the finish line.

6. The average of the two fastest times was calculated as the final performance measure for Part A.

Part B: 500-m Ergometer row test

Part B was conducted in the afternoon of the same day as Part A. Twenty minutes after the completion of Section A, athletes were required to row at maximal effort on an ergometer pre-set to a 500-meter distance. The time to complete the 500-meter row was recorded by designated personnel.

Part C: Composite test (50-m Run + 750-m Ergometer Row + 50-m Run)

Part C was administered on the morning following the day of Part B. The test protocol for each cycle was as follows: athletes first performed a 50-meter straight sprint, then immediately boarded an ergometer and rowed 750 m at maximal effort, followed by a 50-meter sprint back to the starting line.

Specific procedures

1. A stopwatch was positioned at the starting line of the 50-meter sprint to record the total time.

2. Assistants pre-set the ergometer to a 750-meter distance and ensured the ergometer display remained active throughout the test.

3. The test commenced at the starting line: the starter raised the flag, and the stopwatch was activated when the starter dropped the flag and called out “Go”.

4. The stopwatch was stopped when the athlete crossed the original starting line.

Each athlete completed four independent cycles of the above protocol, with a 15-minute recovery interval between consecutive cycles. The total time across the four cycles was used as the final performance metric for Section C.

### Statistical analysis

Data were organized and archived using Microsoft Excel 2021, while all subsequent statistical analyses were performed using IBM SPSS Statistics 26.0 Prior to conducting the main statistical analyses, the Shapiro–Wilk test was applied to verify the normality of the data distribution. A one-way repeated measures analysis of variance (ANOVA) was then used to examine the differences between the within-subject factor (T1, T2, T3). For the repeated measures analysis of variance (ANOVA), the Mauchly’s test of sphericity was first conducted. If the test result indicated that the sphericity assumption was satisfied (*P* > 0.05),the of the one-way of the one-way ANOVA were adopted directly; if the sphericity assumption was violated (*P* < 0.05), the Greenhouse-Geisser correction was applied to adjust the degrees of freedom. Bonferroni correction was implemented for *post-hoc* pairwise comparisons to control for Type I error associated with multiple comparisons. To explore the relationships between variables, correlation analysis was performed. After normality testing, the Pearson product-moment correlation coefficient was used for normally distributed data, while the Spearman’s rank correlation coefficient was used for non-normally distributed data. To investigate the intrinsic associations between the magnitudes of changes, the percentage changes of each indicator from T1 to T3 were calculated, and the correlations among these percentage changes were further analyzed.

**Table 3:** Testing indicators.

Indicator	T1	T2	T3	Within-group comparison (*P*-value)	Effect size (ES)
Back Squat (kg)	120.8 ± 21.9	128.7 ± 19.5	141.9 ± 19.7	T1-T2:*P* < 0.01 T2-T3:*P* < 0.001 T1-T3:*P* < 0.001	T1-T2:ES = 0.38 T2-T3:ES = 0.67 T1-T3:ES = 1.01
Bench Press (kg)	74.4 ± 19.6	82.5 ± 20	92.8 ± 23	T1-T2:*P* < 0.001 T2-T3:*P* < 0.001 T1-T3:*P* < 0.001	T1-T2:ES = 0.40 T2-T3:ES = 0.47 T1-T3:ES = 0.86
Deadlift (kg)	133.0 ± 22.3	139.4 ± 22.1	152.9 ± 20.1	T1-T2:*P* < 0.001 T2-T3:*P* < 0.001 T1-T3:*P* < 0.001	T1-T2:ES = 0.28 T2-T3:ES = 0.63 T1-T3:ES = 0.93
Bench pull	82.8 ± 16.0	89.9 ± 16.3	102.2 ± 20.1	T1-T2:*P* < 0.001 T2-T3:*P* < 0.001 T1-T3:*P* < 0.001	T1-T2:ES = 0.43 T2-T3:ES = 0.67 T1-T3:ES = 1.06
CMJ height(cm)	39.7 ± 6.2	38.8 ± 5.7	41.9 ± 5.4	T1-T2:*P* < 0.05 T2-T3:*P* < 0.001 T1-T3:*P* < 0.001	T1-T2:ES = −0.15 T2-T3:ES = 0.55 T1-T3:ES = 0.37
CMJ Peak Power (W)	3,947.3 ± 588.4	3,873.7 ± 525.6	4,036.1 ± 490.2	T1-T2:*P* < 0.05 T2-T3:*P* < 0.01 T1-T3:*P* > 0.05	T1-T2:ES = −0.13 T2-T3:ES = 0.31 T1-T3:ES = 0.16
50-m Sprint(s)	7.54 ± 0.64	7.5 ± 0.6	7.28 ± 0.6	T1-T2:*P* > 0.05 T2-T3:*P* < 0.001 T1-T3:*P* < 0.001	T1-T2:ES = 0 T2-T3:ES = −0.36 T1-T3:ES = −0.42
500-m Ergometer(s)	94.8 ± 7.8	92.8 ± 7.8	91.1 ± 7.2	T1-T2:*P* < 0.001 T2-T3:*P* < 0.01 T1-T3:*P* < 0.001	T1-T2:ES = −0.26 T2-T3:ES = −0.23 T1-T3:ES = −0.49
Comprehensive Test (50-m Sprint + 750-m Ergometer + 50-m Sprint) (s)	129.3 ± 9.6	124.8 ± 9.1	119.6 ± 8.6	T1-T2:*P* < 0.001 T2-T3:*P* < 0.001 T1-T3:*P* < 0.001	T1-T2:ES = −0.48 T2-T3:ES = −0.59 T1-T3:ES = −1.1

The correlation strength was defined as follows: trivial < 0.10, small 0.10–0.29, moderate 0.30–0.49, large 0.50–0.69, very large 0.70–0.89, and nearly perfect ≥ 0.9 (Nóbrega et al., 2018). The level of statistical significance was set at *P* < 0.05 (statistically significant) and *P* < 0.01 (highly statistically significant). The Cohen’s d was used to standardize the effect size (ES), where ES was categorized as small (0.2–0.49), moderate (0.5–0.79), and large (≥0.8) (Brien, Browne & Earls, 2020). For performance indicators measured by completion time (*e.g.*, 50-m sprint, 500-m ergometer test, composite test), negative effect size (ES) values and negative correlation coefficients (r) indicate a reduction in completion time, reflecting an improvement in performance.

## Results

All participants completed the 14-week training intervention, with no dropouts or injuries reported during the study period. Detailed data on all testing indicators of the athletes at the three time points (T1, T2, T3) are presented in Tables 2 and 3, and a general overview of the key findings is provided as follows:

### Maximal strength

**Figure 1:**
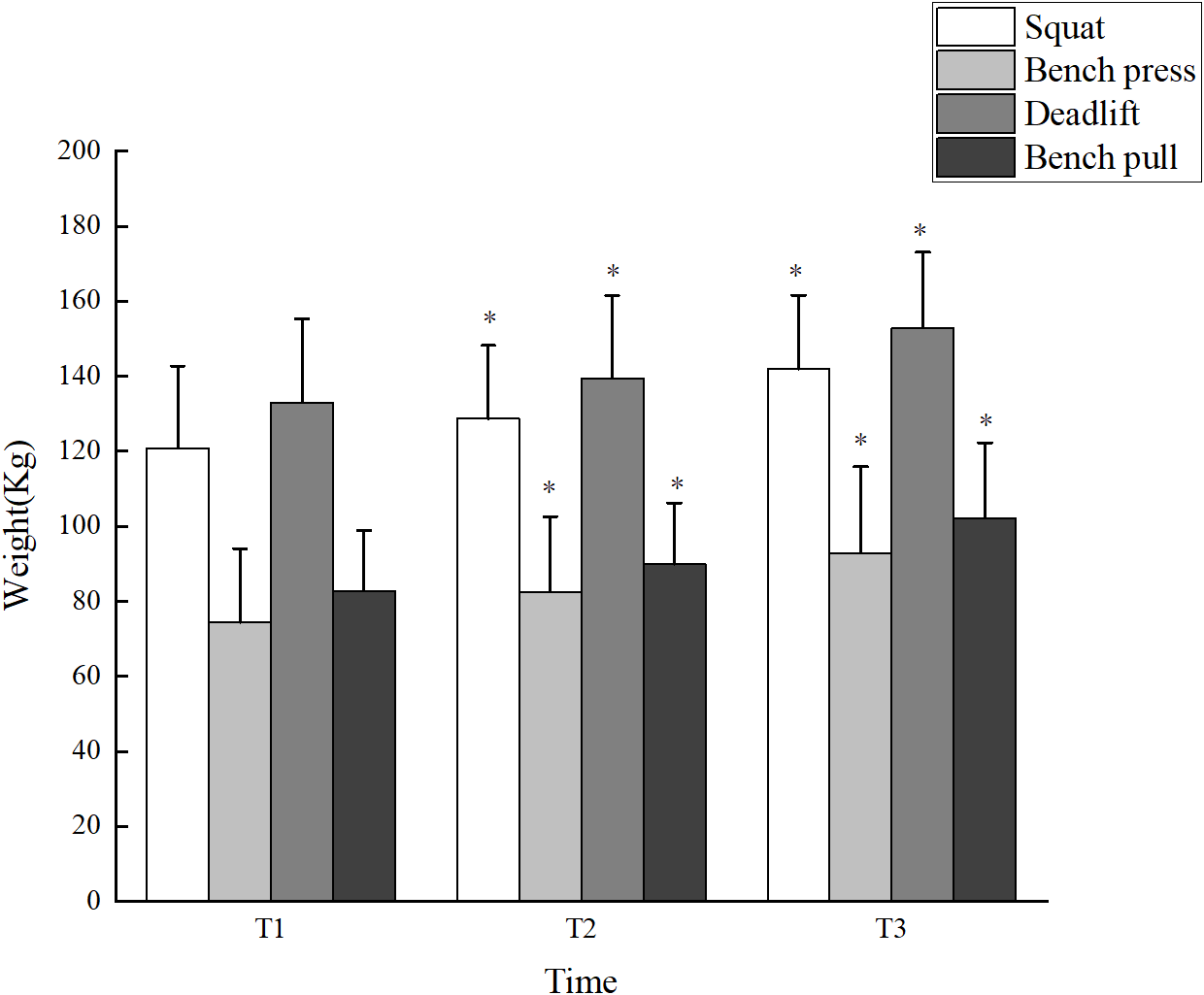
Maximum force variation at different stages.

Significant increases in maximal strength were observed in athletes following 14 weeks of periodized training. As shown in Table 2, from the baseline (T1) to the end of the intervention (T3), the 1-repetition maximum (1RM) values for all four indicators (back squat, bench press, deadlift, and bench pull) exhibited extremely significant increases (see Fig. 1). Notably, the improvement in maximal strength occurred primarily between T2 (end of the hypertrophy phase) and T3 (end of the maximal strength phase), during which the increase in effect size was the most pronounced.

### CMJ and 50-meter sprint

A significant increase in CMJ height was observed from T1 to T3 (*P* < 0.001, ES = 0.37). A slight non-significant decrease was observed from T1 to T2 (*P* > 0.05, ES = − 0.15), while the most significant improvement occurred from T2 to T3 (*P* < 0.001, ES = 0.55). A key observation was that CMJ peak power did not show a statistically significant change throughout the 14 week intervention (T1–T3; *P* > 0.05, ES = 0.16). Specifically, a slight decrease was noted from T1 to T2 (*P* < 0.05, ES= − 0.13); although a significant difference was detected from T2 to T3 (*P* < 0.01, ES = 0.31), the effect size remained small (see Table 2). Similar to CMJ height, the 50-m sprint performance exhibited a significant improvement from T1 to T3, but with a small effect size (*P* < 0.001, ES = −0.26). No significant change was found between T1 and T2 (*P* > 0.05, ES = 0); in contrast, a significant improvement was observed from T2 to T3 (*P* < 0.001, ES= − 0.36), with detailed data presented in Table 2.

### Specialized indicators

Both the 500-meter ergometer performance and the composite test performance (50-meter sprint + 750-meter ergometer + 50-meter sprint) were significantly improved after the intervention.

Specifically, the 500-meter ergometer performance showed significant improvements across all time intervals: T1 to T3 (*P* < 0.001, ES = −0.49), T1 to T2 (*P* < 0.001, ES = −0.26), and T2 to T3 (*P* < 0.01, ES = −0.23) (note: negative ES values indicate reduced completion time, consistent with performance improvement).

**Figure 2:**
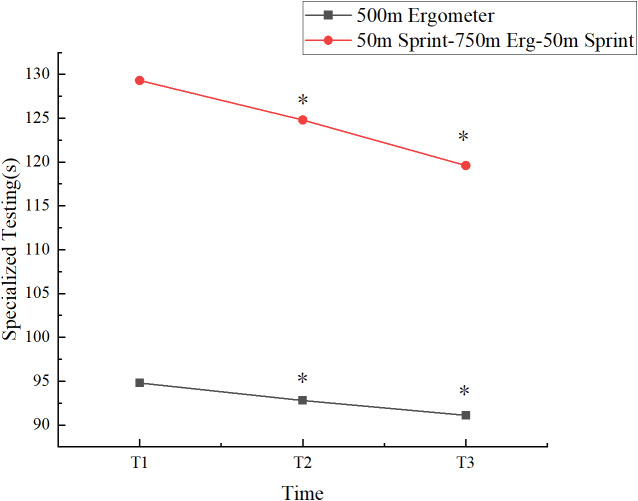
Changes in special indicators.

The comprehensive test (50-meter sprint + 750-meter ergometer + 50-meter sprint) exhibited a greater magnitude of improvement, with significant enhancements observed in T1 to T3 (*P* < 0.001, ES = −1.1), T1 to T2 (*P* < 0.001, ES = −0.48), and T2 to T3 (*P* < 0.001, ES = −0.59) (see Fig. 2 for specific changes).

### Correlation analysis

When the scores of specialized indicators (Test B, Test C) were expressed as Z-scores, the maximal strength (Test B: *P* < 0.001, *r* = −0.862; Test C: *P* < 0.001, *r* = −0.891), (Test B: *P* < 0.001, *r* = −0.898; Test C: *P* < 0.001, *r* = −0.862), (Test B: *P* < 0.001, *r* = −0.910; Test C: *P* < 0.001, *r* = −0.886); 50-meter sprint performance (Test B: *P* < 0.001, *r* = 0.818; Test C: *P* < 0.01, *r* = 0.897), (Test B: *P* < 0.001, *r* = 0.849; Test C: *P* < 0.001, *r* = 0.929), (Test B: *P* < 0.001, *r* = 0.849; Test C: *P* < 0.001, *r* = 0.908); CMJ height (Test B: *P* < 0.01, *r* = −0.785; Test C: *P* < 0.001, *r* = −0.898), (Test B: *P* < 0.001, *r* = −0.801; Test C: *P* < 0.001, *r* = 0.888) (Test B: *P* < 0.01, *r* = −0.794; Test C: *P* < 0.001, *r* = −0.896); and CMJ peak power (Test B: *P* < 0.001, *r* = −0.879; Test C: *P* < 0.001, *r* = −0.760), (Test B: *P* < 0.001, *r* = −0.900; Test C: *P* < 0.001, r = −0.808), (Test B: *P* < 0.001, *r* = −0.888; Test C: *P* < 0.001, *r* = −0.839) all exhibited very large to nearly perfect correlations with these specialized indicators across the three time segments (T1, T2, T3).

During the T1–T3 period, maximal strength (*P* = 0.021, *r* = −0.656) and CMJ height (P = 0.059, *r* = −0.559) showed large correlations with Test B performance. For Test C, a moderate correlation was observed with the 50-meter sprint performance (*P* = 0.103, *r* = 0.493).

**Figure 3:**
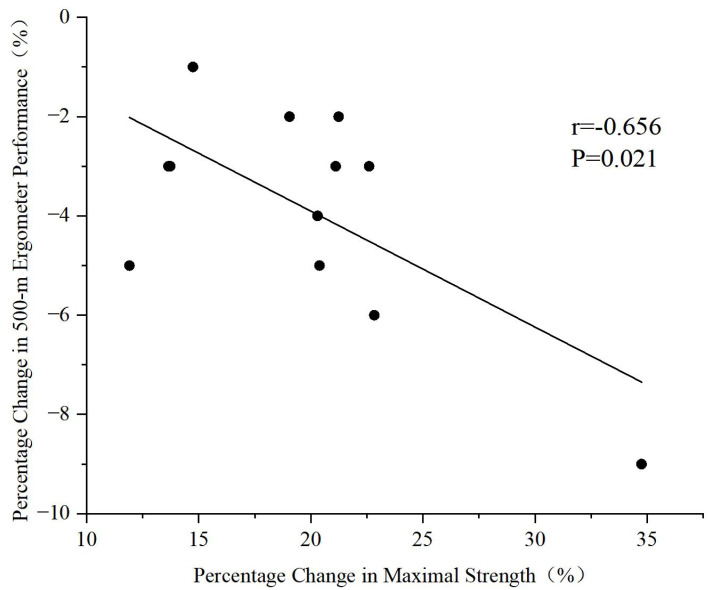
Correlation between improvements in maximal strength and 500-m ergometer performance after 14 weeks of training.

When the segmented indicators (T1–T3) were converted to percentage form for correlation analysis with specialized indicators, the following results were obtained: during the T1–T3 phase, maximal strength (*P* = 0.021, *r* = −0.650) and CMJ height (*P* = 0.059, *r* = −0.550) exhibited large correlations with the 500-meter ergometer performance (see Fig. 3); meanwhile, the 50-meter sprint performance (*P* = 0.103, *r* = 0.493) showed a moderate correlation with the specialized Test C.

## Discussion

This study aimed to investigate the effects of a 14-week movement tempo-based strength periodization program on maximal strength, power, and sport-specific indicators in high-level coastal rowing athletes. The key findings are as follows: (a) From T1 to T3, significant improvements were observed in maximal strength, CMJ height, 50-meter sprint performance, and sport-specific indicators. (b) No significant change in CMJ peak power was detected throughout the intervention period.

This asymmetrical development pattern of strength-height-power reveals the specific adaptation of different dimensions of power, and provides a new perspective for understanding the event-specific demands of coastal rowing. (c) The increase in maximal strength was significantly correlated with the improvement in 500-meter ergometer performance. As an exploratory applied longitudinal intervention study in the emerging Olympic sport of coastal rowing, these results fill critical gaps in existing strength and rowing literature. First, unlike most tempo-based training studies that focus on single contraction phases (eccentric or concentric) or non-elite populations, this is the first study to apply Triphasic-inspired “eccentric-isometric-concentric” tempo sequencing to elite coastal rowers, providing empirical evidence for structured tempo training in this understudied athlete group. Second, compared with traditional flat-water rowing research, this study addresses the unique physical demands of coastal rowing (integrating beach sprint, open-water rowing, and buoy turns) and confirms that tempo-based periodization can enhance sport-specific performance in this distinct discipline. Third, the observed asymmetrical “strength-height-power” adaptation pattern adds new knowledge to the understanding of power development, demonstrating that force-dominant training (rather than velocity-dominant) aligns with the event-specific needs of coastal rowing—an insight not previously reported in rowing-related strength training literature.

These results demonstrate strong associations between the 14-week tempo-based strength periodization program and observed improvements in muscle strength and sport-specific performance.

### Maximal strength and lower limb power

Muscle hypertrophy serves as the foundation for maximal strength (Schoenfeld, 2010; Comfort et al., 2023; Zamparo, Minetti & Di Prampero, 2002; Balshaw et al., 2022; Balshaw et al., 2017), and in a comprehensive periodized strength training program,muscle hypertrophy typically constitutes the initial phase of the cycle (Bompa & Carko, 2019). During the first 6 weeks of training, to achieve progressive overload, the number of exercise sets and specialized training methods were gradually increased over time. This aligns with the established dose–response relationship between training volume and muscle hypertrophy in resistance training (Schoenfeld, Ogborn & Krieger, 2017). Although no muscle hypertrophy-related assessment indicators were included, all athletes exhibited visually observable changes in body composition after 6 weeks of training. In the strength test conducted in the 7th week, significant improvements were observed across all exercises (back squat, bench press, bench pull, and deadlift), which could be attributed to increases in muscle mass and optimization of movement technique. The maximal increase in muscle strength (+11.3%) was achieved during the T2–T3 phase (*i.e.,* the three-phase accumulation phase).

From a training methodology perspective, strength improvement first stems from a systematic periodized plan. Periodized training treats the development of various athletic capacities as an integrated whole, arranging training content in a systematic and cyclical manner in accordance with the objective laws of load adaptation (Matveyev, 2005). From a physiological standpoint, high-load strength training induces neural adaptations, which in turn alter the motor unit recruitment patterns of agonist,antagonist,and synergist muscle groups (Santos et al., 2023). On the other hand, the phase-specific differentiation of concentric, eccentric, and isometric contractions meets the requirement for specific adaptations of muscles to different contraction modes: for instance, tempo control during the eccentric phase is associated with potential changes in neuromuscular coordination efficiency and stretch-shortening cycle (SSC) performance (Mike et al., 2017; Segers et al., 2022; Wilk et al., 2019); pauses during the isometric phase can enhance force output at critical joint angles (Tran, Docherty & Behm, 2006); and accelerated contractions during the concentric phase optimize the force-velocity relationship (Hatfield et al., 2006; González-Badillo et al., 2014; Wilk et al., 2020). This partially aligns with the view of Dietz & Pearson (2012), who proposed that neural pathways may respond differently to stimuli during distinct muscle contraction phases. However, this proposal lacks rigorous empirical validation, as it is difficult to track neural adaptive changes induced by various variables in real-world training processes (Pearcey et al., 2021).

Although the athletes in this study demonstrated significant improvements in maximal strength, the magnitude of improvement did not meet expectations. Due to unavoidable external factors, no intervention could be implemented on sport-specific training. In addition to strength training, the 14-week program included extensive aerobic long-distance rowing on the ergometer. It is well-documented that strength training enhances muscle strength and neuromuscular coordination, whereas aerobic training focuses on improving oxidative capacity. The extreme states of these two adaptations are physiologically incompatible, as a negative correlation exists between skeletal muscle cross-sectional area and mitochondrial oxidative capacity (Van der Zwaard et al., 2018; Van Wessel et al., 2010). This interference effect likely reduced the magnitude of strength improvement induced by the three-phase periodized training. In particular, male athletes, who have a lower proportion of type I muscle fibers, experience more pronounced residual fatigue when combining endurance and strength training, which further impairs strength gains (Leveritt et al., 1999). It is widely recognized that maximal strength is a key driver of high power output, and a strong correlation exists between the two (Haff & Nimphius, 2012; Stone et al., 2024; Comfort, Bullock & Pearson, 2012; Comfort, Haigh & Matthews, 2012; Loturco et al., 2013). Therefore, improving maximal strength is a critical component of programs aimed at maximizing power development. In the present study, an increase in CMJ height was observed following the 14-week training program, a finding that may reflect associations between adaptive responses to the tempo-based periodization training and improvements in CMJ height. Interestingly, despite the significant improvement in CMJ height, no significant increase in CMJ peak power was observed in any of the athletes. This contradicts the findings of most studies, which report a strong correlation between CMJ height and peak power (Aragón-Vargas & Gross, 1997; Barker, Harry & Mercer, 2018; Dowling & Vamos, 1993; Markovic et al., 2014). However, a study by Linthorne (2021) offers a new perspective: according to the formula, peak power (Ppeak) = instantaneous ground reaction force (Fpeak) × instantaneous velocity (Vpeak), power itself is the product of “force” and “velocity”. In contrast, CMJ height (h) is determined by takeoff velocity (Vt0), and this physical relationship inherently links height and power through velocity. Most studies derive the strong correlation between the two based on this formula. It is important to note, however, that during the concentric propulsive phase of the CMJ (from the countermovement to take off from the lowest point), velocity increases almost linearly with the position of the body’s center of mass. This means that “velocity at the moment of peak power (Vpeak)” and “takeoff velocity (Vt0)” change almost synchronously. Since jump height (h) is entirely determined by Vt0, CMJ height and peak power exhibit an almost perfect correlation.

However, this correlation is essentially a result of the “perfect correlation between velocity and height” being “transferred” to power *via* the calculation of “power = force × velocity”, rather than an independent strong causal relationship between power itself and height. Furthermore, Young, Cormack & Crichton (2011) argued that there is no significant correlation between CMJ height and peak power, as the two metrics measure distinct characteristics.

In summary, whether a strong correlation exists between CMJ height and peak power is influenced by testing methods, equipment, and calculation formulas. Based on the results of this study, the authors tend to conclude that CMJ height and peak power reflect different athletic traits of the participants. Specifically, due to the long ground contact time during the takeoff phase of the CMJ, this phase can be categorized as “slow power” (Young, Cormack & Crichton, 2011). This characteristic is particularly similar to the start and acceleration phases of rowing (launch phase) and sprinting (50-meter start and acceleration). Both the 50-meter sprint acceleration and ergometer launch phases require athletes to generate substantial force over an extended support time. On the other hand, during the extension phase of the CMJ, the full extension of the ankle, knee, and hip joints maximizes movement velocity, making this phase more representative of “fast power” (Young, Cormack & Crichton, 2011). This characteristic resembles the latter half of the leg drive phase in rowing. This explanation is logically consistent, as the proportion of power training in the 14-week program was relatively low, which likely resulted in no significant improvement in the athletes’ ankle-knee-hip extension capacity. In addition, jump strategy is one of the key factors influencing CMJ metrics (Flanagan & Comyns, 2008). During the CMJ test, coaches instructed athletes to jump as high as possible. While this instruction is not inappropriate, it may have led some athletes to extend ground contact time to generate greater impulse, thereby increasing jump height.

### Sport-specific indicators

As a composite sport integrating beach sprinting, open-water rowing, and technical buoy turning, coastal rowing’s core demands for sport-specific performance include the ability to transition between high-intensity scenarios, start efficiency in high-resistance beach environments, and power output during rowing with heavy hulls. This is inherently different from traditional flat-water rowing, which features a “single rowing scenario.” The three specialized tests designed in this study (Test A: 50-m beach sprint, Test B: 500-m ergometer, Test C: 50-m sprint + 750-m ergometer + 50-m sprint) were constructed based on the practical competition demands of coastal rowing. The changes in their results require in-depth analysis combining the phase-specific characteristics of the training intervention and the sport’s physiological mechanisms; meanwhile, it is necessary to reference indirect field studies (*e.g.*, beach sports, traditional short-distance rowing) to fill the gap in direct research on coastal rowing.

For competitive sports, the improvement of all physical fitness components aims to enhance sport-specific performance. After 14 weeks of strength training, significant improvements were observed in all sport-specific indicators (Tests A, B, and C). Previous studies have shown that CMJ height is the best predictor of 0–30 m sprint performance (Markström & Olsson, 2013), as both movements involve the coordinated movement of the hip, knee, and ankle joints, and hip extension is critical for sprint speed (McMahon et al., 2017). Although the 50-m sprint performance showed significant improvement over 14 weeks, whether it is suitable as an indicator for evaluating horizontal sprint ability in coastal rowing requires further discussion. This is because sprints in actual competitions take place on sand, and the hardness/softness of the beach surface varies across different coastal areas. The hardness/softness of the beach surface directly determines the ground contact time during sprinting, which is a key factor affecting running speed. Therefore, future research should further explore appropriate horizontal sprint assessment indicators for coastal rowing.

The improvements in Test B and Test C stemmed from three aspects. First, the vary large correlation between maximal strength and sport-specific ergometer indicators (*r* = −0.8 to −0.9) reflects a strong association between these variables—thus, the improvement in maximal strength was strongly associated with the enhanced ergometer performance, and may represent a potential contributing factor to the observed sport-specific performance changes. Existing studies on flat-water rowing have shown that the power contribution of different body segments during rowing follows a clear priority: the leg extension phase contributes approximately 50% of the total rowing power, trunk rotation contributes about 1/3, and arm movement contributes less than 1/5 (Tachibana et al., 2007). Additionally, other studies have confirmed that lower limb strength test results exhibit moderate to high correlations with 2,000-m ergometer performance (*r* = −0.54 to ∼−0.68) (Lawton, Cronin & McGuigan, 2011; Gee et al., 2011). Although the aforementioned studies focused on flat-water rowing, the logic of core techniques (*e.g.*,the force generation sequence of “leg drive–trunk lean–arm pull” and core stability control) is consistent between ergometer rowing and open-water rowing. More importantly, coastal rowing is characterized by “shorter race duration and heavier hull weight,” which places higher demands on athletes to generate maximal force in a short period to overcome resistance. Therefore, the conclusion that “lower limb strength dominates rowing power” can be applied to coastal rowing.

## Limitations

The following limitations should be considered when interpreting the findings of this study:

No control group was established, which was limited by the practical constraints of training in professional sports teams.

The sample size was relatively limited. Although this study included high-level coastal rowing athletes available under the current circumstances, the relatively small sample size may have constrained statistical power, potentially affecting the generalizability of the results.

Environmental factors (*e.g.*, temperature) and increased task familiarity may also have confounded the observed results.

The validity of the sport-specific assessment indicators requires further verification. As an emerging Olympic sport, coastal rowing lacks recognized gold-standard testing protocols. While the sport-specific tests designed in this study strived to simulate real competition demands, their reliability and validity still need confirmation through more rigorous and large-scale studies.

## Conclusions and Recommendations

As an exploratory 14-week applied longitudinal intervention for the emerging Olympic sport of coastal rowing, participation in the tempo-based periodized strength training program was associated with significant improvements in muscle strength and sport-specific performance in well-trained coastal rowers. These observed adaptive changes highlight the potential applied value of structured tempo control in strength periodization for this athlete population, while the causal efficacy remains to be confirmed in future controlled studies. It should be noted that alternative factors such as training maturation and seasonal effects may have contributed to these improvements, which is a limitation inherent to the single-group design.

The significant improvements in 500-m ergometer performance and comprehensive test (Test C) performance suggest that the observed strength gains were associated with enhanced sport-specific output capacity. Additionally, the increase in CMJ height coupled with no significant change in CMJ peak power supports our interpretation of an asymmetrical adaptation pattern of “strength-height-power”—an observational insight rather than a demonstrated physiological mechanism. This pattern, we propose, reflects the program’s emphasis on “force-dominant” rather than “velocity-dominant” adaptations, which aligns with the specific demands of coastal rowing.

Future research should further explore strength-velocity or velocity-strength oriented transfer training following the strength-focused phase, and examine its optimizing effects on sport-specific indicators and the force-velocity curve. On the other hand, the improvement in sport-specific performance partially stems from technical optimization and familiarity with the testing protocol (*e.g.*, proficiency in mounting/dismounting the ergometer in Test C). Future studies should design controlled trials or incorporate covariate analysis to account for these confounding factors.

##  Supplemental Information

10.7717/peerj.21376/supp-1 Supplemental Information 1 Raw data

10.7717/peerj.21376/supp-2 Supplemental Information 2 Statistical analysis data (SPSS)

## Additional Information and Declarations

### Data Availability

The following information was supplied regarding data availability: The raw data is available in the Supplemental Files.

## References

[ref-1] Adrián VM, José OP, Markel GR (2021). Effect of repetition duration—total and in different muscle actions—on the development of strength, power, and muscle hypertrophy: a systematic review. Strength & Conditioning Journal.

[ref-2] Aragón-Vargas LF, Gross MM (1997). Kinesiological factors in vertical jump performance: differences among individuals. Journal of Applied Biomechanics.

[ref-3] Balshaw TG, Massey GJ, Maden-Wilkinson TM, Lanza MB, Folland JP (2022). Effect of long-term maximum strength training on explosive strength, neural, and contractile properties. Medicine & Science in Sports & Exercise.

[ref-4] Balshaw TG, Massey GJ, Maden-Wilkinson TM, Morales-Artacho AJ, McKeown A, Appleby CL, Folland JP (2017). Changes in agonist neural drive, hypertrophy and pre-training strength all contribute to the individual strength gains after resistance training. European Journal of Applied Physiology.

[ref-5] Barker LA, Harry JR, Mercer JA (2018). Relationships between countermovement jump ground reaction forces and jump height, reactive strength index, and jump time. Journal of Strength and Conditioning Research.

[ref-6] Bazyler CD, Abbott HA, Bellon CR, Taber CB, Stone MH (2015). Strength training for endurance athletes: theory to practice. Strength and Conditioning Journal.

[ref-7] Behm S, Jacobs MW, Schumann M (2025). Does maximum strength predict rowing performance in elite female rowers?. International Journal of Sports Physiology and Performance.

[ref-8] Bompa TO, Buzzichelli CA (2019). Periodization theory and methodolgy of training.

[ref-9] Brien JO, Browne D, Earls D (2020). The effects of different types of eccentric overload training on strength, speed, power and change of direction in female basketball players. Journal of Functional Morphology and Kinesioly.

[ref-10] Comfort P, Bullock N, Pearson SJ (2012). A comparison of maximal squat strength and 5-, 10-, and 20-meter sprint times, in athletes and recreationally trained men. Journal of Strength and Conditioning Research.

[ref-11] Comfort P, Haff GG, Suchomel TJ, Soriano MA, Pierce KC, Hornsby WG, Haff EE, Sommerfield LM, Chavda S, Morris SJ, Fry AC, Stone MH (2023). National strength and conditioning association position statement on weightlifting for sports performance. Journal of Strength and Conditioning Research.

[ref-12] Comfort P, Haigh A, Matthews MJ (2012). Are changes in maximal squat strength during preseason training reflected in changes in sprint performance in rugby league players?. Journal of Strength and Conditioning Research.

[ref-13] Dietz C, Peterson B (2012). Triphasic training: a systematic approach to elite speed and explosive strength performance.

[ref-14] Dowling JJ, Vamos L (1993). Identification of kinetic and temporal factors related to vertical jump performance. Journal of Applied Biomechanics.

[ref-15] Flanagan EP, Comyns TM (2008). The use of contact time and the reactive strength index to optimize fast stretch-shortening cycle training. Strength & Conditioning Journal.

[ref-16] Gee TI, Olsen PD, Berger NJ, Golby J, Thompson KG (2011). Strength and conditioning practices in rowing. Journal of Strength and Conditioning Research.

[ref-17] González-Badillo JJ, Rodríguez-Rosell D, Sánchez-Medina L, Gorostiaga EM, Pareja-Blanco F (2014). Maximal intended velocity training induces greater gains in bench press performance than deliberately slower half-velocity training. European Journal of Sport Science.

[ref-18] Haff GG, Nimphius S (2012). Training principles for power. Strength and Conditioning Journal.

[ref-19] Hartmann H, Wirth K, Keiner M, Mickel C, Sander A, Szilvas E (2015). Short-term periodization models: effects on strength and speed-strength performance. Sports Medicine.

[ref-20] Hatfield DL, Kraemer WJ, Spiering BA, Häkkinen K, Volek JS, Shimano T, Spreuwenberg LP, Silvestre R, Vingren JL, Fragala MS, Gómez AL, Fleck SJ, Newton RU, Maresh CM (2006). The impact of velocity of movement on performance factors in resistance exercise. Journal of Strength and Conditioning Research.

[ref-21] Lawton TW, Cronin JB, McGuigan MR (2011). Strength testing and training of rowers: a review. Sports Medicine.

[ref-22] Ledergerber R, Jacobs MW, Roth R, Schumann M (2023). Contribution of different strength determinants on distinct phases of Olympic rowing performance in adolescent athletes. European Journal of Sport Science.

[ref-23] Leveritt M, Abernethy PJ, Barry BK, Logan PA (1999). Concurrent strength and endurance training. A review. Sports Medicine.

[ref-24] Linthorne NP (2021). The correlation between jump height and mechanical power in a countermovement jump is artificially inflated. Sports Biomechanics.

[ref-25] Loturco I, Ugrinowitsch C, Roschel H, Lopes Mellinger A, Gomes F, Tricoli V, Gonzáles-Badillo JJ (2013). Distinct temporal organizations of the strength- and power-training loads produce similar performance improvements. Journal of Strength and Conditioning Research.

[ref-26] Markovic S, Mirkov DM, Nedeljkovic A, Jaric S (2014). Body size and countermovement depth confound relationship between muscle power output and jumping performance. Human Movement Science.

[ref-27] Markström JL, Olsson CJ (2013). Countermovement jump peak force relative to body weight and jump height as predictors for sprint running performances: (in) homogeneity of track and field athletes?. Journal of Strength and Conditioning Research.

[ref-28] Songping Y, Matveyev (2005). The theory of competitive sports.

[ref-29] McMahon JJ, Murphy S, Rej SJE, Comfort P (2017). Countermovement-jump-phase characteristics of senior and academy Rugby league players. International Journal of Sports Physiology and Performance.

[ref-30] Michal W, Tufano JJ, Adam Z (2020). The influence of movement tempo on acute neuromuscular, hormonal, and mechanical responses to resistance exercise—a mini review. Journal of Strength and Conditioning Research.

[ref-31] Mike JN, Cole N, Herrera C, Van Dusseldorp T, Kravitz L, Kerksick CM (2017). The effects of eccentric contraction duration on muscle strength, power production, vertical jump, and soreness. Journal of Strength and Conditioning Research.

[ref-32] Mujika I, Halson S, Burke LM, Balagué G, Farrow D (2018). An integrated, multifactorial approach to periodization for optimal performance in individual and team sports. International Journal of Sports Physiology and Performance.

[ref-33] Nóbrega SR, Barroso R, Ugrinowitsch C, Da Costa JLF, Alvarez IF, Barcelos C, Libardi CA (2018). Self-selected *vs.* fixed repetition duration: effects on number of repetitions and muscle activation in resistance-trained men. Journal of Strength and Conditioning Research.

[ref-34] Nugent FJ, Flanagan EP, Wilson F, Warrington GD (2020). Strength and conditioning for competitive rowers. Strength and Conditioning Journal.

[ref-35] Pearcey GEP, Alizedah S, Power KE, Button DC (2021). Chronic resistance training: is it time to rethink the time course of neural contributions to strength gain?. European Journal of Applied Physiology and Occupational Physiology.

[ref-36] Pearson J, Wadhi T, Barakat C, Aube D, Schoenfeld BJ, Andersen JC, Barroso R, Ugrinowitsch C, De Souza EO (2022). Does varying repetition tempo in a single-joint lower body exercise augment muscle size and strength in resistance-trained men?. Journal of Strength and Conditioning Research.

[ref-37] Pereira PEA, Motoyama YL, Esteves GJ, Quinelato WC, Azevedo P (2016). Resistance training with slow speed of movement is better for hypertrophy and muscle strength gains than fast speed of movement. International Journal of Applied Exercise Physiology.

[ref-38] Santos PDG, Vaz JR, Correia J, Neto T, Pezarat-Correia P (2023). Long-term neurophysiological adaptations to strength training: a systematic review with cross-sectional studies. Journal of Strength and Conditioning Research.

[ref-39] Schoenfeld BJ (2010). The mechanisms of muscle hypertrophy and their application to resistance training. Journal of Strength and Conditioning Research.

[ref-40] Schoenfeld BJ, Ogborn D, Krieger JW (2017). Dose–response relationship between weekly resistance training volume and increases in muscle mass: a systematic review and meta-analysis. Journal of Sports Sciences.

[ref-41] Segers N, Waldron M, Howe LP, Patterson SD, Moran J, Jones B, Kidgell DJ, Tallent J (2022). Slow-speed compared with fast-speed eccentric muscle actions are detrimental to jump performance in elite soccer players in-season. International Journal of Sports Physiology and Performance.

[ref-42] Shibata K, Takizawa K, Nosaka K, Mizuno M (2021). Effects of prolonging eccentric phase duration in parallel back-squat training to momentary failure on muscle cross-sectional area, squat one repetition maximum, and performance tests in university soccer players. Journal of Strength and Conditioning Research.

[ref-43] Stasinaki AN, Zaras N, Methenitis S, Bogdanis G, Terzis G (2019). Rate of force development and muscle architecture after fast and slow velocity eccentric training. Sports.

[ref-44] Stone MH, Hornsby G, Mizuguchi S, Sato K, Gahreman D, Duca M, Carroll K, Ramsey MW, Stone ME, Haff GG (2024). The use of free weight squats in sports: a narrative review-squatting movements, adaptation, and sports performance: physiological. Journal of Strength and Conditioning Research.

[ref-45] Stone MH, Hornsby WG, Haff GG, Fry AC, Suarez DG, Liu J, Gonzalez-Rave JM, Pierce KC (2021). Periodization and block periodization in sports: emphasis on strength-power training—a provocative and challenging narrative. Journal of Strength and Conditioning Research.

[ref-46] Tachibana K, Yashiro K, Miyazaki J, Ikegami Y, Higuchi M (2007). Muscle cross-sectional areas and performance power of limbs and trunk in the rowing motion. Sports Biomechanics.

[ref-47] Thiele D, Prieske O, Lesinski M, Granacher U (2020). Effects of equal volume heavy-resistance strength training *versus* strength endurance training on physical fitness and sport-specific performance in young elite female rowers. Frontiers in Physiology.

[ref-48] Tran QT, Docherty D, Behm D (2006). The effects of varying time under tension and volume load on acute neuromuscular responses. European Journal of Applied Physiology and Occupational Physiology.

[ref-49] Van der Zwaard S, Van der Laarse WJ, Weide G, Bloemers FW, Hofmijster MJ, Levels K, Noordhof DA, De Koning JJ, De Ruiter CJ, Jaspers RT (2018). Critical determinants of combined sprint and endurance performance: an integrative analysis from muscle fiber to the human body. FASEB Journal.

[ref-50] Van Wessel T, De Haan A, Van der Laarse WJ, Jaspers RT (2010). The muscle fiber type–fiber size paradox: hypertrophy or oxidative metabolism?. European Journal of Applied Physiology and Occupational Physiology.

[ref-51] Walker S, Trezise J, Haff GG, Newton RU, Häkkinen K, Blazevich AJ (2020). Increased fascicle length but not patellar tendon stiffness after accentuated eccentric-load strength training in already-trained men. European Journal of Applied Physiology and Occupational Physiology.

[ref-52] Wilk M, Golas A, Krzysztofik M, Nawrocka M, Zajac A (2019). The effects of eccentric cadence on power and velocity of the bar during the concentric phase of the bench press movement. Journal of Sports Science and Medicine.

[ref-53] Wilk M, Krzysztofik M, Drozd M, Zajac A (2020). Changes of power output and velocity during successive sets of the bench press with different duration of eccentric movement. International Journal of Sports Physiology and Performance.

[ref-54] Young W, Cormack S, Crichton M (2011). Which jump variables should be used to assess explosive leg muscle function?. International Journal of Sports Physiology and Performance.

[ref-55] Zamparo P, Minetti AE, Di Prampero PE (2002). Interplay among the changes of muscle strength, cross-sectional area and maximal explosive power: theory and facts. European Journal of Applied Physiology.

