# Supplementary material for: Can tempo-based strength periodization training improve performance in coastal rowers? A 14-week longitudinal study
Source: PeerJ. 2026 Jun 18;14:e21376. doi: 10.7717/peerj.21376 (PMC13283366; doi:10.7717/peerj.21376)
Supplement: Supplemental Information 2 [file peerj-14-21376-s002.pdf]

1. Correlation Analysis Z-Score

A.Correlations between **Z-score 500m** and Maximal Strength, 50m Sprint, CMJ Peak Power, CMJ Height (T1, T2, T3)

Correlations

		Z 500m Z五百米	Maximal Strength 最大力量总和（T1）	50m Sprint 五十米冲刺（T1）	CMJ Peak power CMJ峰值功率（T1）	CMJ height CMJ高度（T1）	Maximal Strength 最大力量总和（T2）	50m Sprint 五十米冲刺（T2）	CMJ Peak power CMJ峰值功率（T2）	CMJ height CMJ高度（T2）	Maximal Strength 最大力量总和（T3）	50m Sprint 五十米冲刺（T3）	CMJ height CMJ高度（T3）
Z五百米 Z 500m	Pearson Correlation	1	-.862**	.818**	-.879**	-.785**	-.898**	.818**	-.900**	-.801**	-.910**	.849**	-.794**
	Sig. (2-tailed)		.000	.001	.000	.002	.000	.001	.000	.002	.000	.000	.002
	N	12	12	12	12	12	12	12	12	12	12	12	12
最大力量总和（T1） Maximal Strength	Pearson Correlation	-.862**	1	-.807**	.783**	.816**	.985**	-.791**	.829**	.785**	.972**	-.791**	.760**
	Sig. (2-tailed)	.000		.002	.003	.001	.000	.002	.001	.002	.000	.002	.004
	N	12	12	12	12	12	12	12	12	12	12	12	12
五十米冲刺（T1） 50m Sprint	Pearson Correlation	.818**	-.807**	1	-.614*	-.955**	-.817**	.977**	-.685*	-.942**	-.848**	.972**	-.950**
	Sig. (2-tailed)	.001	.002		.034	.000	.001	.000	.014	.000	.000	.000	.000
	N	12	12	12	12	12	12	12	12	12	12	12	12
CMJ峰值功率（T1） CMJ Peak power	Pearson Correlation	-.879**	.783**	-.614*	1	.622*	.832**	-.656*	.989**	.639*	.849**	-.701*	.621*
	Sig. (2-tailed)	.000	.003	.034		.031	.001	.020	.000	.025	.000	.011	.031
	N	12	12	12	12	12	12	12	12	12	12	12	12
CMJ高度（T1） CMJ height	Pearson Correlation	-.785**	.816**	-.955**	.622*	1	.806**	-.975**	.691*	.982**	.832**	-.959**	.989**
	Sig. (2-tailed)	.002	.001	.000	.031		.002	.000	.013	.000	.001	.000	.000
	N	12	12	12	12	12	12	12	12	12	12	12	12
最大力量总和（T2） Maximal Strength	Pearson Correlation	-.898**	.985**	-.817**	.832**	.806**	1	-.789**	.874**	.787**	.992**	-.807**	.755**
	Sig. (2-tailed)	.000	.000	.001	.001	.002		.002	.000	.002	.000	.002	.005
	N	12	12	12	12	12	12	12	12	12	12	12	12
五十米冲刺（T2） 50m Sprint	Pearson Correlation	.818**	-.791**	.977**	-.656*	-.975**	-.789**	1	-.718**	-.964**	-.827**	.987**	-.981**
	Sig. (2-tailed)	.001	.002	.000	.020	.000	.002		.009	.000	.001	.000	.000
	N	12	12	12	12	12	12	12	12	12	12	12	12
CMJ峰值功率（T2） CMJ Peak power	Pearson Correlation	-.900**	.829**	-.685*	.989**	.691*	.874**	-.718**	1	.717**	.891**	-.758**	.688*
	Sig. (2-tailed)	.000	.001	.014	.000	.013	.000	.009		.009	.000	.004	.013
	N	12	12	12	12	12	12	12	12	12	12	12	12
CMJ高度（T2） CMJ height	Pearson Correlation	-.801**	.785**	-.942**	.639*	.982**	.787**	-.964**	.717**	1	.810**	-.945**	.983**
	Sig. (2-tailed)	.002	.002	.000	.025	.000	.002	.000	.009		.001	.000	.000
	N	12	12	12	12	12	12	12	12	12	12	12	12
最大力量总和（T3） Maximal Strength	Pearson Correlation	-.910**	.972**	-.848**	.849**	.832**	.992**	-.827**	.891**	.810**	1	-.854**	.793**
	Sig. (2-tailed)	.000	.000	.000	.000	.001	.000	.001	.000	.001		.000	.002
	N	12	12	12	12	12	12	12	12	12	12	12	12
五十米冲刺（T3） 50m Sprint	Pearson Correlation	.849**	-.791**	.972**	-.701*	-.959**	-.807**	.987**	-.758**	-.945**	-.854**	1	-.970**
	Sig. (2-tailed)	.000	.002	.000	.011	.000	.002	.000	.004	.000	.000		.000
	N	12	12	12	12	12	12	12	12	12	12	12	12
CMJ高度（T3） CMJ height	Pearson Correlation	-.794**	.760**	-.950**	.621*	.989**	.755**	-.981**	.688*	.983**	.793**	-.970**	1
	Sig. (2-tailed)	.002	.004	.000	.031	.000	.005	.000	.013	.000	.002	.000	
	N	12	12	12	12	12	12	12	12	12	12	12	12

**. Correlation is significant at the 0.01 level (2-tailed).

Note: CMJ peak power at T3 was not normally distributed; therefore, Spearman's correlation was used for the analysis.

Nonparametric Correlations

Correlations

			Z 500m Erg Z五百米	CMJ peak power T3 CMJ峰值功率（T3）
Spearman's rho	Z五百米 Z 500m Erg	Correlation Coefficient	1.000	-.888**
		Sig. (2-tailed)	.	.000
		N	12	12
	CMJ峰值功率（T3） CMJ peak power T3	Correlation Coefficient	-.888**	1.000
		Sig. (2-tailed)	.000	.
		N	12	12

**. Correlation is significant at the 0.01 level (2-tailed).

B.Correlations between **Z-score Composite Test** and Maximal Strength, 50m Sprint, CMJ Peak Power, CMJ Height (T1, T2, T3)

Correlations

Z Composite test		Z Composite test Z复合	Maximal Strength T1 最大力量总和（T1）	50m Sprint T1 五十米冲刺（T1）	CMJ peak power T1 CMJ峰值功率（T1）	CMJ height T1 CMJ高度（T1）	Maximal Strength T2 最大力量总和（T2）	50m Sprint T2 五十米冲刺（T2）	CMJ peak power T2 CMJ峰值功率（T2）	CMJ height T2 CMJ高度（T2）	Maximal Strength T3 最大力量总和（T3）	50m Sprint T3 五十米冲刺（T3）	CMJ height T3 CMJ高度（T3）
Z复合	Pearson Correlation	1	-.891**	.897**	-.760**	-.898**	-.862**	.929**	-.808**	-.888**	-.886**	.908**	-.896**
	Sig. (2-tailed)		.000	.000	.004	.000	.000	.000	.001	.000	.000	.000	.000
	N	12	12	12	12	12	12	12	12	12	12	12	12
最大力量总和（T1） Maximal Strength T1	Pearson Correlation	-.891**	1	-.807**	.783**	.816**	.985**	-.791**	.829**	.785**	.972**	-.791**	.760**
	Sig. (2-tailed)	.000		.002	.003	.001	.000	.002	.001	.002	.000	.002	.004
	N	12	12	12	12	12	12	12	12	12	12	12	12
五十米冲刺（T1） 50m Sprint T1	Pearson Correlation	.897**	-.807**	1	-.614*	-.955**	-.817**	.977**	-.685*	-.942**	-.848**	.972**	-.950**
	Sig. (2-tailed)	.000	.002		.034	.000	.001	.000	.014	.000	.000	.000	.000
	N	12	12	12	12	12	12	12	12	12	12	12	12
CMJ峰值功率（T1） CMJ peak power T1	Pearson Correlation	-.760**	.783**	-.614*	1	.622*	.832**	-.656*	.989**	.639*	.849**	-.701*	.621*
	Sig. (2-tailed)	.004	.003	.034		.031	.001	.020	.000	.025	.000	.011	.031
	N	12	12	12	12	12	12	12	12	12	12	12	12
CMJ高度（T1） CMJ height T1	Pearson Correlation	-.898**	.816**	-.955**	.622*	1	.806**	-.975**	.691*	.982**	.832**	-.959**	.989**
	Sig. (2-tailed)	.000	.001	.000	.031		.002	.000	.013	.000	.001	.000	.000
	N	12	12	12	12	12	12	12	12	12	12	12	12
最大力量总和（T2） Maximal Strength T2	Pearson Correlation	-.862**	.985**	-.817**	.832**	.806**	1	-.789**	.874**	.787**	.992**	-.807**	.755**
	Sig. (2-tailed)	.000	.000	.001	.001	.002		.002	.000	.002	.000	.002	.005
	N	12	12	12	12	12	12	12	12	12	12	12	12
五十米冲刺（T2） 50m Sprint T2	Pearson Correlation	.929**	-.791**	.977**	-.656*	-.975**	-.789**	1	-.718**	-.964**	-.827**	.987**	-.981**
	Sig. (2-tailed)	.000	.002	.000	.020	.000	.002		.009	.000	.001	.000	.000
	N	12	12	12	12	12	12	12	12	12	12	12	12
CMJ峰值功率（T2） CMJ peak power T2	Pearson Correlation	-.808**	.829**	-.685*	.989**	.691*	.874**	-.718**	1	.717**	.891**	-.758**	.688*
	Sig. (2-tailed)	.001	.001	.014	.000	.013	.000	.009		.009	.000	.004	.013
	N	12	12	12	12	12	12	12	12	12	12	12	12
CMJ高度（T2） CMJ height T2	Pearson Correlation	-.888**	.785**	-.942**	.639*	.982**	.787**	-.964**	.717**	1	.810**	-.945**	.983**
	Sig. (2-tailed)	.000	.002	.000	.025	.000	.002	.000	.009		.001	.000	.000
	N	12	12	12	12	12	12	12	12	12	12	12	12
最大力量总和（T3） Maximal Strength T3	Pearson Correlation	-.886**	.972**	-.848**	.849**	.832**	.992**	-.827**	.891**	.810**	1	-.854**	.793**
	Sig. (2-tailed)	.000	.000	.000	.000	.001	.000	.001	.000	.001		.000	.002
	N	12	12	12	12	12	12	12	12	12	12	12	12
五十米冲刺（T3） 50m Sprint T3	Pearson Correlation	.908**	-.791**	.972**	-.701*	-.959**	-.807**	.987**	-.758**	-.945**	-.854**	1	-.970**
	Sig. (2-tailed)	.000	.002	.000	.011	.000	.002	.000	.004	.000	.000		.000
	N	12	12	12	12	12	12	12	12	12	12	12	12
CMJ高度（T3） CMJ height T3	Pearson Correlation	-.896**	.760**	-.950**	.621*	.989**	.755**	-.981**	.688*	.983**	.793**	-.970**	1
	Sig. (2-tailed)	.000	.004	.000	.031	.000	.005	.000	.013	.000	.002	.000	
	N	12	12	12	12	12	12	12	12	12	12	12	12

**. Correlation is significant at the 0.01 level (2-tailed).

Note: CMJ peak power at T3 was not normally distributed; therefore, Spearman's correlation was used for the analysis.

Nonparametric Correlations

Correlations

Z Composite test			Z Composite test Z复合	CMJ peak power T3 CMJ峰值功率（T3）
Spearman's rho	Z复合	Correlation Coefficient	1.000	-.839**
		Sig. (2-tailed)	.	.001
		N	12	12
	CMJ峰值功率（T3） CMJ peak power T3	Correlation Coefficient	-.839**	1.000
		Sig. (2-tailed)	.001	.
		N	12	12

**. Correlation is significant at the 0.01 level (2-tailed).

2. Correlation Analysis percentage

Correlations

Maximal Strength T1-T3		Maximal Strength T1-T3 前至后，最大力量百分比	CMJ height T1-T3 前至后，CMJ高度百分比	CMJ peak power T1-T3 前至后，CMJ峰值百分比	50m sprint T1-T3 前至后，五十米冲刺百分比	500m Erg T1-T3 前至后，五百米测功仪百分比	Composite test T1-T3 前至后，专项测试百分比
前至后，最大力量百分比	Pearson Correlation	1	.680*	-.118	-.324	-.656*	-.231
	Sig. (2-tailed)		.015	.715	.305	.021	.470
	N	12	12	12	12	12	12
CMJ height T1-T3 前至后，CMJ高度百分比	Pearson Correlation	.680*	1	.012	-.296	-.559	-.198
	Sig. (2-tailed)	.015		.970	.350	.059	.538
	N	12	12	12	12	12	12
CMJ peak power T1-T3 前至后，CMJ峰值百分比	Pearson Correlation	-.118	.012	1	.453	.343	.115
	Sig. (2-tailed)	.715	.970		.139	.275	.721
	N	12	12	12	12	12	12
50m sprint T1-T3 前至后，五十米冲刺百分比	Pearson Correlation	-.324	-.296	.453	1	.239	.493
	Sig. (2-tailed)	.305	.350	.139		.454	.103
	N	12	12	12	12	12	12
500m Erg T1-T3 前至后，五百米测功仪百分比	Pearson Correlation	-.656*	-.559	.343	.239	1	.554
	Sig. (2-tailed)	.021	.059	.275	.454		.062
	N	12	12	12	12	12	12
Composite test T1-T3 前至后，专项测试百分比	Pearson Correlation	-.231	-.198	.115	.493	.554	1
	Sig. (2-tailed)	.470	.538	.721	.103	.062	
	N	12	12	12	12	12	12

*. Correlation is significant at the 0.05 level (2-tailed).

Note: The following segmented data were not normally distributed, so Spearman's correlation was applied.

Correlations

			Maximal Strength T1-T2<br>前至中，最大力量百分比	CMJ height T1-T2<br>前至中，CMJ高度百分比	CMJ peak power T1-T2<br>前至中，CMJ峰值功率百分比	50m Sprint T1-T2<br>前至中，五十米冲刺百分比	500m Erg T1-T2<br>前至中，五百米测功仪百分比	Composite test T1-T2<br>前至中，复合测试百分比
Spearman's rho	前至中，最大力量百分比<br>Maximal Strength T1-T2	Correlation Coefficient	1.000	.442	-.326	.200	-.100	-.416
		Sig. (2-tailed)	.	.151	.302	.533	.757	.178
		N	12	12	12	12	12	12
	前至中，CMJ高度百分比<br>CMJ height T1-T2	Correlation Coefficient	.442	1.000	.202	.188	.236	-.820**
		Sig. (2-tailed)	.151	.	.529	.559	.461	.001
		N	12	12	12	12	12	12
	前至中，CMJ峰值功率百分比<br>CMJ peak power T1-T2	Correlation Coefficient	-.326	.202	1.000	.401	.467	.018
		Sig. (2-tailed)	.302	.529	.	.196	.126	.955
		N	12	12	12	12	12	12
	前至中，五十米冲刺百分比<br>50m Sprint T1-T2	Correlation Coefficient	.200	.188	.401	1.000	.261	.134
		Sig. (2-tailed)	.533	.559	.196	.	.413	.677
		N	12	12	12	12	12	12
	前至中，五百米测功仪百分比<br>500m Erg T1-T2	Correlation Coefficient	-.100	.236	.467	.261	1.000	-.406
		Sig. (2-tailed)	.757	.461	.126	.413	.	.190
		N	12	12	12	12	12	12
	前至中，复合测试百分比<br>Composite test T1-T2	Correlation Coefficient	-.416	-.820**	.018	.134	-.406	1.000
		Sig. (2-tailed)	.178	.001	.955	.677	.190	.
		N	12	12	12	12	12	12

**. Correlation is significant at the 0.01 level (2-tailed).

Correlations

Maximal Strength T2-T3

			Maximal Strength T2-T3<br>中至后，最大力量百分比	CMJ height T2-T3<br>中至后，CMJ高度百分比	CMJ peak power T2-T3<br>中至后，CMJ峰值功率百分比	50m Sprint T2-T3<br>中至后，五十米冲刺百分比	500m Erg T2-T3<br>中至后，五百米测功仪百分比	Composite test T2-T3<br>中至后，复合测试百分比
Spearman's rho	中至后，最大力量百分比	Correlation Coefficient	1.000	.468	.306	-.374	-.175	-.119
		Sig. (2-tailed)	.	.125	.334	.231	.586	.714
	CMJ height T2-T3	N	12	12	12	12	12	12
	中至后，CMJ高度百分比	Correlation Coefficient	.468	1.000	.306	-.214	-.356	.382
		Sig. (2-tailed)	.125	.	.334	.503	.256	.221
	CMJ peak power T2-T3	N	12	12	12	12	12	12
	中至后，CMJ峰值功率百分比	Correlation Coefficient	.306	.306	1.000	.259	-.037	.418
		Sig. (2-tailed)	.334	.334	.	.416	.909	.176
	50m Sprint T2-T3	N	12	12	12	12	12	12
	中至后，五十米冲刺百分比	Correlation Coefficient	-.374	-.214	.259	1.000	.257	-.071
		Sig. (2-tailed)	.231	.503	.416	.	.420	.825
	500m Erg T2-T3	N	12	12	12	12	12	12
	中至后，五百米测功仪百分比	Correlation Coefficient	-.175	-.356	-.037	.257	1.000	.116
		Sig. (2-tailed)	.586	.256	.909	.420	.	.719
	Composite test T2-T3	N	12	12	12	12	12	12
	中至后，复合测试百分比	Correlation Coefficient	-.119	.382	.418	-.071	.116	1.000
		Sig. (2-tailed)	.714	.221	.176	.825	.719	.
		N	12	12	12	12	12	12
